# Flammability, Toxicity, and Microbiological Properties of Polyurethane Flexible Foams

**DOI:** 10.3390/ma17143517

**Published:** 2024-07-16

**Authors:** Arkadiusz Głowacki, Przemysław Rybiński, Grzegorz Czerwonka, Witold Żukowski, Ulugbek Zakirovich Mirkhodjaev, Monika Żelezik

**Affiliations:** 1 Institute of Chemistry, The Jan Kochanowski University, 25-406 Kielce, Poland; 2 Institute of Biology, Jan Kochanowski University, 25-406 Kielce, Poland; grzegorz.czerwonka@ujk.edu.pl; 3 Faculty of Chemical Engineering and Technology, Cracow University of Technology, Warszawska 24, 31-155 Cracow, Poland; witold.zukowski@pk.edu.pl; 4 Department of Biophysics, National University of Uzbekistan, Tashkent 100095, Uzbekistan; u.z.mirkhodjaev@gmail.com; 5 Institute of Geography and Environmental Sciences, Jan Kochanowski University, 25-406 Kielce, Poland; monika.zelezik@ujk.edu.pl

**Keywords:** PUR composites, smoke emission, toxicometric index, phosphinates, organic phosphates, fire hazard, fire retardants

## Abstract

The aim of the research was to investigate the influence of calcium phosphinate (HPCA) and aluminum phosphinate (HPAL) in synergistic systems with organophosphorus compounds, i.e., diphenylcresyl phosphate (CDP) and trichloropropyl phosphate (TCPP), on the thermal stability, flammability, smoke density, and emission of toxic gases during the thermal decomposition of polyurethane (PUR) foams. Thermogravimetric analysis (TGA), along with cone calorimetry and microcalorimetry, were used to assess the influence of fillers on the thermal stability and flammability of PUR foams. The analysis of toxic gas products was performed with the use of a coupled TG–gas analyzer system. The optical density of gases was measured with the use of a smoke density chamber (SDC). The obtained results showed an increase in thermal stability and a decrease in the flammability of the PUR composites. However, the results regarding smoke and gas emissions, as well as toxic combustion by-products, present ambiguity. On one hand, the applied flame retardant systems in the form of PUR-HPCA-CDP and PUR-HPCA-TCPP led to a reduction in the concentration of CO and HCN in the gas by-products. On the other hand, they clearly increased the concentration of CO_2_, NOx, and smoke emissions. Microbiological studies indicated that the obtained foam material is completely safe for use and does not exhibit biocidal properties.

## 1. Introduction

Polyurethane materials (PUR), due to their potential for mass production, wide range of modifications, and versatile properties, are widely used in many sectors of the industry as well as in everyday life [1,2,3].

An extremely important feature of PUR materials is their ability to be modified by changing the type of raw materials, the relative quantities of the substrates, or the processing conditions. Depending on their chemical composition, the resulting PUR composites can exhibit high mechanical strength, noise- and vibration-damping properties, increased resistance to physico-chemical factors, and resistance to organic solvents and oils [4,5,6]. However, a significant factor limiting the application of polyurethane materials is their low fire resistance. The combustion of PUR foams is accompanied by a high release of heat, as well as the intensive emission of smoke and toxic combustion products [7,8,9,10].

Previous research has unequivocally shown that significant amounts of smoke, CO, and CO_2_ are emitted during the initial phase of PUR thermal decomposition [11]. At higher decomposition temperatures, i.e., above 400 °C, high concentrations of HCN and NO_2_ are additionally released [12,13]. Nitrogen compounds can cause damage to the respiratory and nervous systems, and with prolonged exposure, they can also affect the cardiovascular system. In extreme cases, they may even lead to death [14,15,16]. One of the approaches to enhancing the applicability of PUR (polyurethane) materials involves research aimed at improving their fire resistance, including the use of organophosphorus compounds. A review of the literature indicates that organophosphorus compounds, especially in synergistic systems with mineral or carbon fillers, increase the thermal stability of PUR foam while simultaneously reducing its flammability [17,18,19].

The key mechanism that reduces the fire hazard of polyurethane in the presence of organophosphorus compounds is the formation of a char barrier layer on the surface of the PUR, particularly in the initial stages of thermal decomposition. This barrier prevents the spread of fire both across the surface and into the interior of the polymer [20,21].

In addition to satisfactory fire resistance, PUR materials, due to their widespread contact with the human body, must also be biologically safe. This means they should not contain potentially allergenic or toxic chemical components. To determine whether a composite with the desired functional properties is safe, model in vitro analyses are conducted. These analyses typically involve a wide range of studies using defined cell lines [22,23].

Antibacterial PUR composites are used in hospitals and sanitary wards [24]. Additionally, they can be utilized in the production of everyday materials such as exercise mats, sleeping pads, and upholstery foam [25,26]. Most antibacterial fillers for PUR, in the form of silver, gold particles, or metal oxides, do not exhibit selective biocidal action. Consequently, the antibacterial activity of polymer materials results in the destruction of bacterial cells [27,28]. This effect often leads to the rupture of microorganism cells, releasing toxic cellular components onto the composite surface. These components can be irritating or even toxic due to the presence of numerous enzymes and parts of bacterial cell membranes and walls. The unintentional introduction of these toxins into the human body can have serious consequences, including inflammatory states or even cross-reactions [29,30].

Currently used PUR composites do not exhibit long-term antibacterial properties while maintaining neutrality towards naturally occurring skin bacteria. Therefore, biocompatible PUR materials with long-term effectiveness remain the subject of intensive research.

This article presents the results of studies on the effects of phosphine compounds, also in synergistic systems with organophosphorus compounds, on the properties of PUR composites, with particular emphasis on their fire hazard and biocompatibility.

## 2. Materials and Methods

### 2.1. Materials

**Figure 1:**
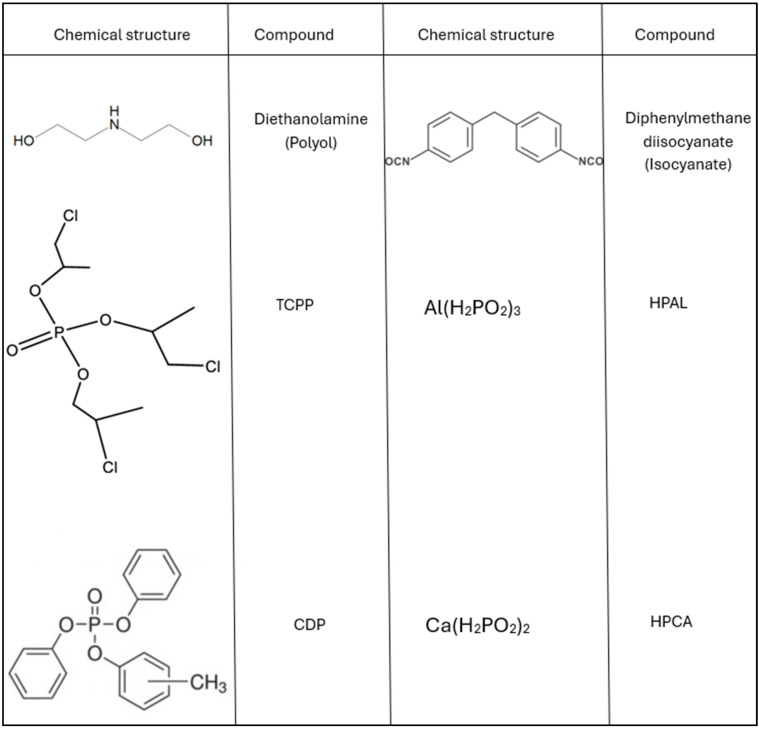
Chemical structure of the components.

The subject of the study was flexible polyurethane foam synthesized using polyol (BASF, Elastoflex W5165/140) and isocyanate (BASF, IZO 135/158) (diphenylmethane diisocyanate-MDI) in a 2:1 ratio. The fillers used were calcium phosphinate (HPAL), aluminum phosphinate (HPCA), trichloropropyl phosphate (TCPP), and diphenylcresyl phosphate (CDP) (Everkrem, Italy) (Figure 1).

#### 2.1.1. Preparation of PUR Composites

The PUR foams were synthesized using a one-step reaction with the two-component polyol-isocyanate in a weight ratio of OH:NCO of 2:1 (Table 1). The obtained PUR composites were conditioned to a constant mass at a temperature of 23 ± 2 °C and humidity not exceeding 50 ± 5%, according to ISO 291 [31]. Conditioning is considered properly conducted if the sample’s mass after 24 h does not exceed an error range of 0.1 g or 0.1%.

#### 2.1.2. Preparation of Bacterial Culture

Microorganism activity studies against PUR composites were conducted with reference bacterial strains: *Staphylococcus aureus* ATCC 6538P [32], *Escherichia coli* ATCC 8739 [33], and *Bacillus subtilis* PCM486 [34]. The bacteria were taken directly from frozen culture (−80 °C in 8% DMSO in LB) onto solid LB medium (5 g yeast extract, 10 g tryptone, 0.5 g NaCl, 1.5 g agar per liter of water). Cultures were incubated for 24 h at 37 °C. The overnight culture was transferred to fresh liquid LB medium and then incubated for 24 h at 37 °C with shaking (160 rpm) (EcoTron, Infors HT). The bacteria were then centrifuged twice using a laboratory centrifuge (5000 rpm, 10 min) and suspended in saline (0.85% NaCl). The bacterial suspension was diluted to an optical density of 1 (OD~1, λ = 550 nm). This is the initial density, assumed to contain between 10^8^ and 10^9^ CFU bacteria.

### 2.2. Methods

#### 2.2.1. Scanning Electron Microscopy

Scanning electron microscopy (SEM) imaging was performed using an Apreo 2 S LoVac microscope (Thermo Fisher Scientific, Waltham, MA 02451, USA) equipped with energy-dispersive X-ray spectroscopy (EDS) detectors: UltraDry (Thermo Fisher Scientific, USA) and Octane Elect (EDAX Ametek GmbH) (Hitachi, Tokyo, Japan) with an accelerating voltage of 2 kV.

#### 2.2.2. Fourier-Transform Infrared Spectroscopy Analysis

Fourier-transform infrared spectroscopy with attenuated total reflection (FTIR-ATR) was conducted using a PerkinElmer Spectrum device (Waltham, MA, USA). The FTIR spectrophotometer was equipped with a diamond ATR crystal, with a single reflection on a ZeSe plate. Measurements were recorded using the PerkinElmer Spectrum software 10 (USA). Spectra were recorded using 4 scans with a resolution of 4 cm^−1^ in the MIR range of 400–4000 cm^−1^ in transmission mode.

#### 2.2.3. Thermal Analysis

Thermal analysis was performed using a Netzsch STA 449 F3 Jupiter analyzer (Selb, Germany) in the temperature range from 25 to 650 °C for samples with a mass of 5 ± 1 mg. The samples were placed in an open Al_2_O_3_ crucible. Measurements were carried out in an air atmosphere with a heating rate of 10 °C/min.

#### 2.2.4. Classification of Microbial Growth—UV-Vis Analysis

The study of microorganism activity was conducted using a method based on changes in UV-VIS light absorption with a Jasco V-660 spectrophotometer (Tokyo 193-0835, Japan). Measurements were taken at a light wavelength of 550 nm. The results were collected in two stages. In the first stage, the initial bacterial suspension sample was measured, with an optical density (OD) of approximately 1. The second stage of the microorganism activity study in the presence of composites involved collecting absorbance results after overnight incubation of the bacterial suspension with an initial OD of ~1 with the PUR, PUR-HPCA-CDP, PUR-HPAL-CDP, PUR-HPAL-TCPP, and PUR-HPCA-TCPP composites. The background for all results was a 0.85% NaCl solution. The activity measurement in the presence of composites was calculated using the following formula:

$$Activity=\frac{AbsMeasured}{AbsInitial}\times 100\%$$

where:

Activity—microbiological activity (%);

AbsMeasured—recorded absorption after the incubation stage of the suspension with PUR composites;

AbsInitial—recorded initial absorption of the bacterial suspension (OD~1).

#### 2.2.5. Toxicity Tests of Gaseous Decomposition Products

The toxicity of gaseous decomposition products of PUR composites was studied using a coupled TG system (Netzsch TG 209 F1 Libra, Selb, Germany) and an FTIR Omega 5 analyzer (Bruker Omega 5, Billerica, MA, USA). The gas analysis was conducted over a temperature range of ΔT = 30–650 °C in an air atmosphere. The heating rate was 10 °C/min. Gas emissions were determined for samples weighing 5 ± 1 mg. FTIR spectra were recorded every 7–8 s (10 scans).

The concentrations of gases (in ppm) were determined using the Opus GA software (version 8.7.14, Hillview, 18103-6046 USA) gases analyzed included CO, CO_2_, HCl, NO, and NO_2_. The conversion of the concentration of gaseous decomposition products expressed in ppm to concentrations expressed in g/m^3^ or mg/m^3^ was performed using the following relationships:

pV=nRT

where:

P—pressure (101,325 Pa);

V—gas volume;

N—number of moles of particles in the gas;

R—gas constant (8314 J/mol·K).

$$E\frac{\mathrm{mg}}{m^{3}}=\frac{\mathrm{ppm}\;\mathrm{mol}\;\mathrm{compound}\;\times \;M\;\times 1000\frac{\mathrm{mg}}{g}}{10^{-6}\;\mathrm{mol}\;\mathrm{gas}}\begin{array}{c} \to \mathrm{mg}\\ \;\;\to {\mathrm{Nm}}^{3} \end{array}$$

$$V=\frac{8.314\frac{J}{\mathrm{mol}\;\times \;K}\times 298\;K\;\times 10^{6}}{101,325\;\mathrm{Pa}}$$

$$E\frac{\mathrm{mg}}{m^{3}}=\frac{\mathrm{ppm}\;\mathrm{mol}\;\mathrm{compound}\;\times \;M\;\times 1000\frac{\mathrm{mg}}{g}}{\;\frac{8.314\frac{J}{\mathrm{mol}\;\times \;K}\times 298\;K\;\times 10^{6}}{101,325\;\mathrm{Pa}}}$$

Shortened conversion to g/m^3^

$$E\frac{g}{m^{3}}=\frac{\mathrm{ppm}\;\times \;M}{{\mathrm{Nm}}^{3}\times 1000\;}$$

The toxicometric index (CITG) was calculated based on the formula [35] (Table 2):

$${\mathrm{CIT}}_{G}=\frac{\mathrm{Es}}{{\mathrm{LC}}_{50}^{30}}\times 0.0805$$

where:

0.0805—toxicity constant according to ISO 5659-2 Annex C [35], according to which 0.1 m^2^ of exposed product emits gases that disperse in a volume of 150 m^3^;

Es—sampled spectrum corresponding to 4 L of emitted gas (sampled emission);

${\mathrm{LC}}_{50}^{30}$—lethal concentration for 50% of the test population over 30 min.

**Table 2:** Gas limit concentration [36].

Determined Gas	CO	CO_2_	HCN	NO_2_	HCl	SO_2_
${\mathrm{LC}}_{50}^{30}$	1380 mg/m^3^	72,000 mg/m^3^	55 mg/m^3^	38 mg/m^3^	75 mg/m^3^	262 mg/m^3^

#### 2.2.6. Flammability: PCFC Microcalorimetry

The flammability of PUR composites was tested using a PCFC (pyrolysis combustion flow calorimetry) microcalorimeter manufactured by Fire Testing Technology Ltd., East Grinstead, UK. The procedure was conducted according to ASTM D 7309 [36]. The pyrolyzer temperature was 650 °C with a heating rate of 1 °C/s, and the combustion chamber temperature was 900 °C. The test was carried out under nitrogen/oxygen conditions (80/20 cm^3^/min). The following parameters were recorded during the test: heat release rate (HRR), maximum heat release rate (HRR_MAX_) (W/g), time to HRR_MAX_ (s), total heat release (HR) (kJ/g), and heat release capacity (HRC) (J/gK).

#### 2.2.7. Flammability: Cone Calorimetry

The fire hazard of PUR composites was evaluated using a cone calorimeter from Fire Testing Technology Ltd. according to PN-EN ISO 5660 [37]. Samples of standardized dimensions 100 × 100 × 50 mm were analyzed in a horizontal position using a heat flux of 35 kW/m^2^. The following parameters were recorded during the test: initial sample mass, sample mass during the test, final sample mass, time to ignition (T_i_), time to sample extinction (T_f-0_), total heat release (THR), effective heat of combustion (EHC), average mass loss rate (MLR), heat release rate (HRR), fire growth rate index (FIGRA), and the maximum average heat release rate (MARHE).

#### 2.2.8. Smoke Density

Smoke density was tested using the smoke density chamber (SDC) according to PN-EN ISO 5659-2 [38]. Samples of standardized dimensions 75 × 75 × 15 mm were tested with a heat flux of 25 kW/m^2^. The following parameters were recorded during the test: initial sample mass, sample mass during the test, final sample mass, maximum specific optical density of smoke (D_sMAX_), optical density at 4 min of testing (Ds(4)), area under the specific optical density curve (VOF4), and light attenuation coefficient after the test.

## 3. Results and Discussion

### 3.1. Surface Morphology and FTIR Analysis of PUR Composites

**Figure 2:**
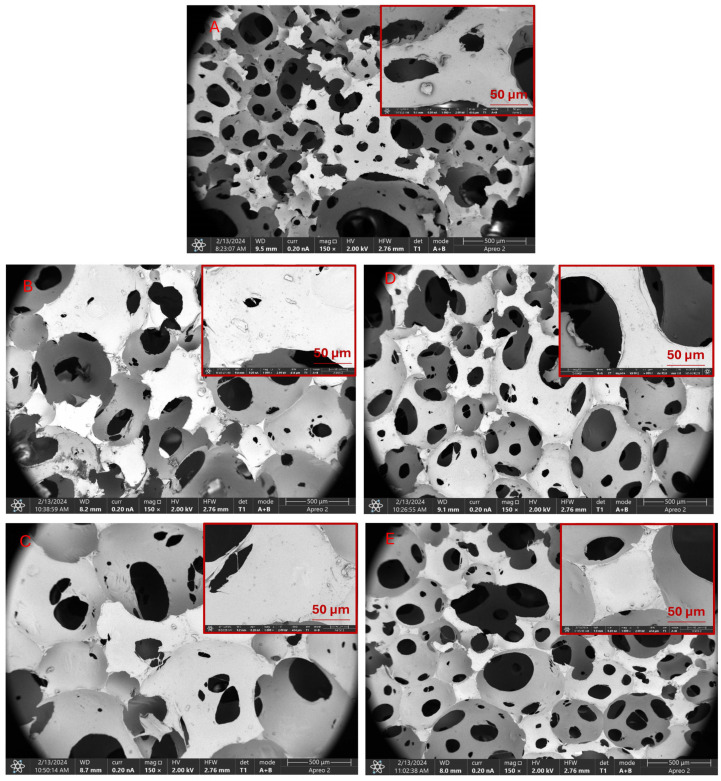
SEM imaging. (**A**) PUR, (**B**) PUR-HPAL-CDP, (**C**) PUR-HPAL-TCPP, (**D**) PUR-HPCA-CDP, (**E**) PUR-HPCA-TCPP.

The spatial structure morphology studies conducted using SEM clearly indicate the porous structure of PUR composites. The introduction of organophosphorus compounds into the PUR matrix, especially in the form of aluminum phosphinate (HPAL), significantly reduces the volume of free spaces in the PUR matrix (PUR-HPAL-TCPP/CDP system) (Figure 2A–C). Based on the obtained SEM images, it can also be concluded that calcium phosphinate (HPCA), in the PUR-HPCA-TCPP/CDP system, reduces the formation of the porous structure of PUR to a much lesser extent compared to the PUR-HPAL-TCPP/CDP system (Figure 2D,E). However, it should be emphasized that in the presence of the PUR-HPCA-TCPP/CDP system, the porosity of the PUR composite is significantly lower compared to the reference composite (Figure 2A,D,E).

The increase in the porosity of the PUR composite structure undoubtedly has a significant impact on improving its elastic properties, and consequently, its ability to dampen vibrations. Highly elastic and inherently porous PUR foams are commonly used for upholstery purposes, including the production of seats for the railway, aviation, and automotive industries [39,40]. Upholstery foams used in the broadly understood public transport industry, in addition to satisfactory performance parameters, such as elasticity and resistance to delamination, must also exhibit appropriate fire hazard parameters, such as reduced flammability, smoke emission, and toxic gas emissions. The fire hazard requirements for polyurethane foams are regulated by appropriate standards [35].

The impact of the porosity of the PUR composite on fire hazard parameters is ambiguous. On the one hand, as the porosity of the composite increases, the amount of polymer material per unit volume of the composite decreases, which may result in some reduction in flammability. On the other hand, the free spaces in PUR are filled with air, which catalyzes the degradation and destruction reactions of the polymer material, especially in the initial stage of its decomposition [41].

The SEM results unequivocally indicate that phosphinate compounds reduce the porosity of the PUR structure. In the presence of aluminum phosphinate, the porosity of PUR foam is lower than in the presence of calcium phosphinate. This is likely directly related to the molecular weight of both compounds. The 23% lower molecular weight of calcium phosphinate (HPCA) compared to aluminum phosphinate (HPAL) causes the polyurethane foam to expand much more easily in the presence of HPCA (Figure 2).

**Figure 3:**
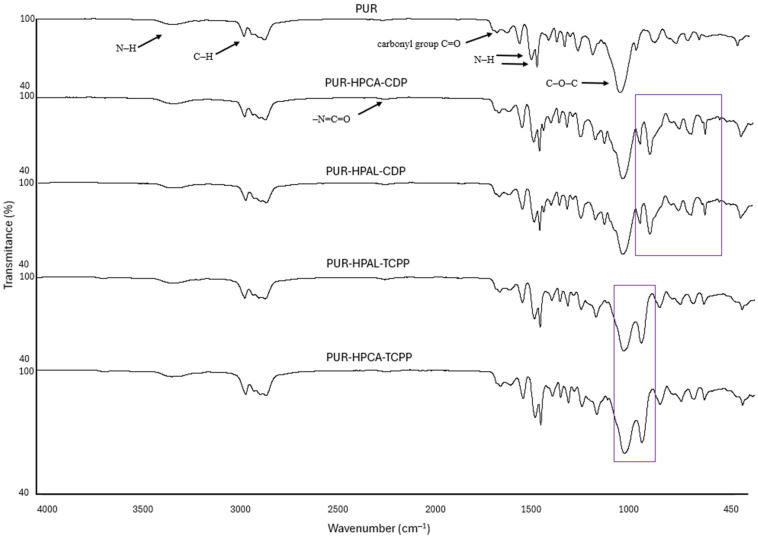
FTIR spectra of PUR composites.

During the polyurethane synthesis reaction, the characteristic disappearance of NCO stretching bands at 2270 cm^−1^ is typically observed. However, in the obtained composites, a minimal peak can still be noticed in this region. The presence of free isocyanate group bonds in the polyurethane composites (PUR-HPCA-CDP, PUR-HPAL-CDP, PUR-HPAL-TCPP, PUR-HPCA-TCPP) suggests the presence of unreacted sites, which may not be adequately filled by the nanofillers [42]. In the IR spectrum of the PUR matrix, broad stretching absorption bands are registered at 3337 cm^−1^, corresponding to stretching vibrations, as well as at 1538 cm^−1^ and 1510 cm^−1^, corresponding to N-H group vibrations. At 1460 and 1260 cm^−1^, signals are present from both symmetric and asymmetric vibrations of the N-C-N group [43]. Vibrations characteristic of the methylene group, i.e., symmetric and asymmetric vibrations, are registered at 2955 and 2851 cm^−1^, respectively. At 1450 cm^−1^, bending vibrations from the C-H group are registered. The IR spectrum of the PUR matrix also shows signals at 1655 cm^−1^ and 1710 cm^−1^ corresponding to stretching vibrations of the carbonyl group and a strong, characteristic band at 1095 cm^−1^ related to stretching vibrations of the C-O-C group (Figure 3) [44,45,46,47].

The FTIR results of the PUR-HPCA-CDP, PUR-HPAL-CDP, PUR-HPAL-TCPP, and PUR-HPCA-TCPP composites did not show significant changes in the IR spectrum compared to the reference PUR matrix. Composites containing a synergistic flame retardant system in the form of HPCA-CDP and HPAL-CDP exhibit band deformations in the range of 1010 cm^−1^ to 600 cm^−1^, including an increase in the band intensity at 956 cm^−1^. For these composites, a new signal was also recorded at 1190 cm^−1^, corresponding to the valence vibrations of the phosphate group. In the case of composites containing HPCA-TCPP and HPAL-TCPP, signal deformations occur at 1230 cm^−1^ and in the range of 700 cm^−1^ to 550 cm^−1^. The recorded changes in the IR spectra of the studied PUR composites may indicate the chemical bonding of organophosphorus compounds with the PUR matrix (Figure 3).

### 3.2. Thermal Analysis and Flammability of PUR Composites

**Figure 4:**
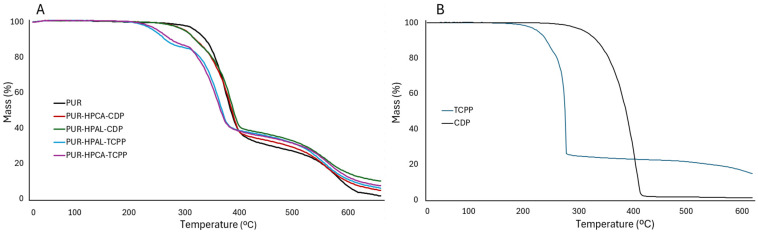
Thermal curves: (**A**) PUR, PUR-HPCA-CDP, PUR-HPAL-CDP, PUR-HPAL-TCPP, PUR-HPCA-TCPP; (**B**) organophosphorus fillers TCPP and CDP.

Thermal analysis results unequivocally indicate that the introduction of hybrid flame retardant systems, HPCA(HPAL)-CDP and HPCA(HPAL)-TCPP, into the PUR foam matrix reduces its thermal stability, expressed by both T_5_ and T_50_ parameters. A significantly greater reduction in the T_5_ and T_50_ values was recorded in the presence of TCPP than CDP (Table 3). It is possible that the carbon–chlorine bonds present at the ends of the alkyl chains of TCPP (Figure 1) undergo homolytic dissociation at temperatures around 200 °C. The produced chlorine radicals participate in interrupting high-energy combustion reactions occurring in the gas phase and may accelerate the degradation and destruction of the polymer matrix. The increased efficiency of thermal degradation processes of the PUR matrix in the presence of TCPP is also confirmed by the maximum decomposition temperature parameter, T_RMAX_. In the presence of TCPP, the T_RMAX_ value of the PUR composite was reduced by 16 °C (PUR-HPAL-TCPP) and 19 °C (PUR-HPCA-TCPP) compared to the unfilled PUR composite. It should also be emphasized that the introduction of CDP, containing large aromatic substituents, into the PUR matrix, in the HPCA(HPAL)-CDP system, results in an increase in the T_RMAX_ parameter value (Figure 4, Table 3).

The fire hazard of PUR composites is primarily characterized by two thermal stability parameters: the rate of thermal decomposition dm/dt and the residue after thermal decomposition P_TD_. It is generally assumed that the lower the dm/dt value, the smaller the amount of volatile, including flammable, decomposition products entering the flame zone. In the case of very low dm/dt values, the flame ceases to be fueled, and consequently, the composite self-extinguishes.

The residue after thermal decomposition, P_TD_, indicates the intensity of processes such as carbonization or cyclization occurring during the thermal decomposition of the composite. These processes directly affect both the thickness and morphology of the boundary layer [48]. A homogeneous, insulating boundary layer is crucial in reducing the fire hazard of the studied PUR composites. With increasing homogeneity and insulation of the boundary layer, both mass transport and heat transfer between the sample and the flame are reduced [49,50].

**Figure 5:**
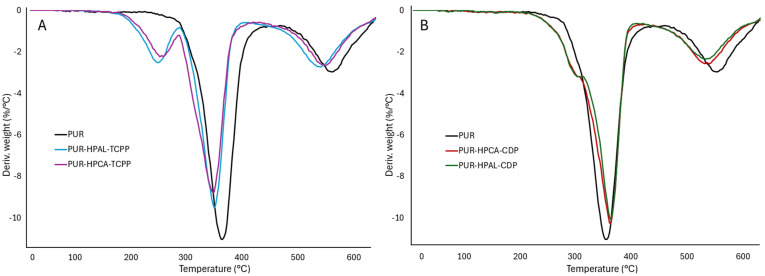
DTG analysis of PUR composites: (**A**) PUR, PUR-HPAL-TCPP, PUR-HPCA-TCPP; (**B**) PUR, PUR-HPCA-CDP, PUR-HPAL-CDP.

Thermal analysis results indicate that the introduction of both the HPCA(HPAL)-CDP and HPCA(HPAL)-TCPP systems into the PUR matrix reduces both the dm/dt and P_TD_ parameters. Therefore, it can be concluded that both studied systems should be effective in reducing the fire hazard of PUR composites (Table 3, Figure 4 and Figure 5).

Diphenyl cresyl phosphate CDP, due to the presence of large aromatic substituents in its structure, exhibits a much greater tendency towards carbonization processes than tris(1-chloro-2-propyl) phosphate, TCPP. The P_TD_ parameter values for both the HPCA(HPAL)-CDP and HPCA(HPAL)-TCPP systems are similar. This indicates that inorganic aluminum (HPAL) and calcium phosphinate (HPCA) significantly influence the residue after thermal decomposition, the P_TD_ parameter, and P_600_ (Table 3, Figure 4 and Figure 5). The obtained research results suggest that the boundary layer formed during the thermal decomposition of the PUR composite is primarily stabilized by inorganic phosphorus compounds (HPAL and HPCA).

**Figure 6:**
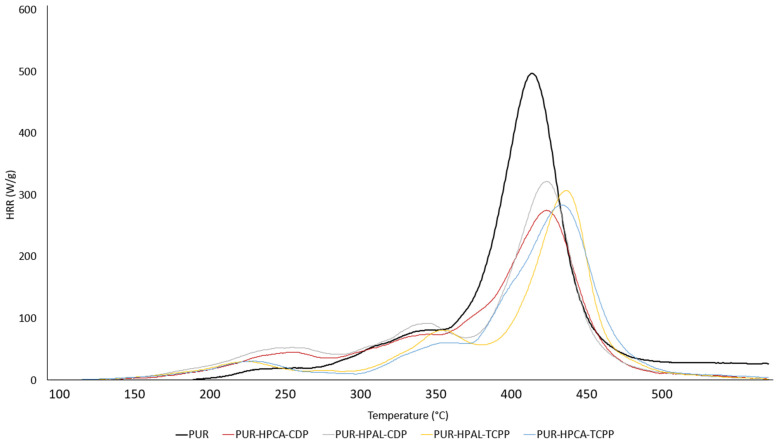
Flammability results of PUR composites obtained using the PCFC microcalorimetry method.

The results obtained using PCFC microcalorimetry clearly indicate that both the HPCA(HPAL)-CDP and HPCA(HPAL)-TCPP systems effectively reduce the flammability of PUR composites (Figure 6, Table 4). Notably, there was nearly a 40% reduction in the HRR_MAX_ parameter for the PUR-HPCA-CDP sample and a 33% reduction in the THR parameter for the PUR-HPCA-TCPP sample. Both the HPCA(HPAL)-CDP and HPCA(HPAL)-TCPP systems show similar effectiveness in reducing the flammability of the studied PUR composites [51,52]. However, it should be noted that in the presence of HPCA(HPAL)-TCPP, the value of the THRR_MAX_ parameter significantly increases. This may indicate that the boundary layer of PUR forms faster, i.e., at a lower temperature range, in the presence of HPCA(HPAL)-TCPP compared to HPCA(HPAL)-CDP (Table 3).

The results obtained using PCFC correlate well with the flammability results obtained using cone calorimetry (Table 4 and Table 5). The fire hazard parameters obtained under real conditions indicate that both the HPCA(HPAL)-CDP and HPCA(HPAL)-TCPP systems reduce the flammability of PUR composites. The tested flame-retardant systems reduce both kinetic parameters such as HRR_MAX_ and MARHE, which indicate fire kinetics, and the FIGRA parameter, which indicates the rate of fire development. It is also worth noting that the tested systems clearly reduce the total heat released, the THR parameter (Table 5).

### 3.3. Smoke Emission and Toxicity of Combustion Products

The results obtained using the SDC (smoke density chamber) method showed that the synergistic flame retardant system used, HPCA(HPAL)-CDP and HPCA(HPAL)-TCPP, has an ambiguous effect on smoke emission during thermal decomposition (Table 6). For samples containing HPCA-CDA, HPAL-CDA, and HPAL-TCPP, an increase in smoke emission, the D_sMAX_ parameter, was recorded. However, for the HPCA-TCPP sample, the amount of smoke emitted during sample decomposition was significantly reduced. It should also be clearly noted that the amount of smoke emitted for all tested samples increases during the first 4 min of thermal decomposition, parameters Ds(4) and VOF_4_ (Table 6).

The increase in smoke emissions during the decomposition of the tested samples undoubtedly results from the presence of organophosphorus compounds in their matrix, which intensify carbonization and cyclization processes. The carbonization processes occurring during the thermal decomposition of PUR composites can cause increased smoke emissions.

**Figure 7:**
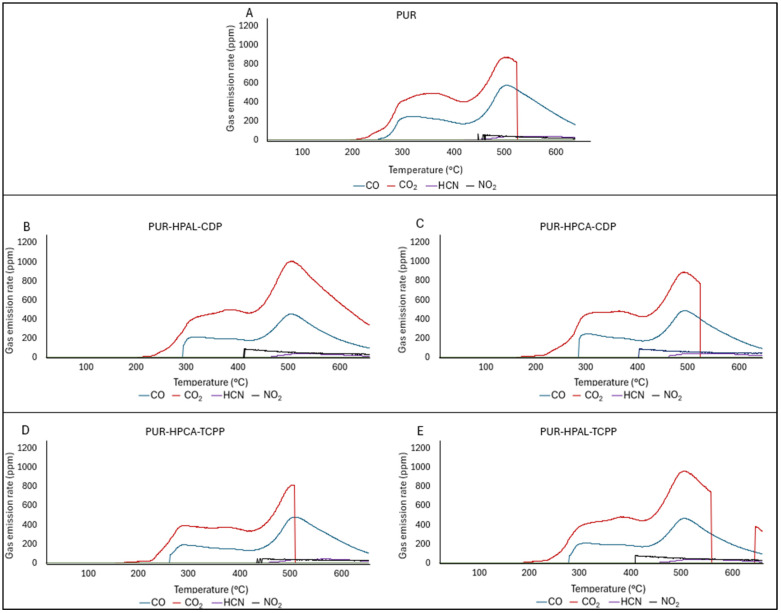
Real-time toxic gas emissions.

The analysis of the toxicity of gaseous decomposition products during the thermal decomposition of the tested PUR composites showed that the flame retardant systems used, HPCA(HPAL)-CDP and HPCA(HPAL)-TCPP, also reduce the concentration of emitted CO and HCN. For the PUR-HPAL-CDP system, the reduction in emitted HCN concentration was as high as 21% (Table 7). However, it should also be noted that the flame retardant systems used caused an increase in emitted CO_2_ concentration and a significant increase in NOx concentration. Only in the PUR-HPCA-TCPP composite can a significant reduction in the emission of toxic gases be observed, which correlates with the results obtained using the smoke density chamber. Consequently, the CIT_G_ toxicometric index for the PUR-HPCA-CDP, PUR-HPCA-TCPP, and PUR-HPAL-TCPP composites turned out to be higher than for the reference PUR material (Table 7). The increase in the CIT_G_ index for the tested composite materials is primarily due to the increase in emitted CO_2_ concentration. The increase in carbon dioxide content in the gaseous decomposition products of PUR thermal decomposition, like the increase in smoke emission, is directly due to the presence of organophosphorus compounds in the PUR matrix. However, it should be clearly noted that the increase in CO_2_ emission is evident only at high temperature values, i.e., above 420 °C (Figure 7, Table 7). This indicates that the emitted CO_2_ is primarily produced as a result of the partial degradation of the carbonized boundary layer.

### 3.4. Analysis of Microbiological Activity

The results of microbiological activity analysis against PUR composites were obtained using the UV-VIS method. The obtained results showed clear changes in the level of bacterial adsorption to the composite surface (Table 8). The bacteria used in the analysis (*Escherichia coli*, *Bacillus subtilis*, and *Staphylococcus aureus*) are reference strains used to assess microorganism activity. Differences in the effectiveness of adsorption from the bacterial suspension to the PUR surface were observed, ranging from 45% to 62% between bacterial species. However, the average degree of bacterial binding to the surface did not show significant differences for PUR-HPCA-CDP 55%, PUR-HPAL-CDP 57.1%, and PUR-HPAL-TCPP 54.6%. The lowest average absorption levels were shown for the original PUR 49% and PUR-HPCA-TCPP 48% (Table 8). It can be observed that composites characterized by smaller pores in SEM imaging (Figure 2), and therefore the largest foam surface, showed the most effective level of bacterial binding to the material (HPAL) (Table 8).

**Figure 8:**
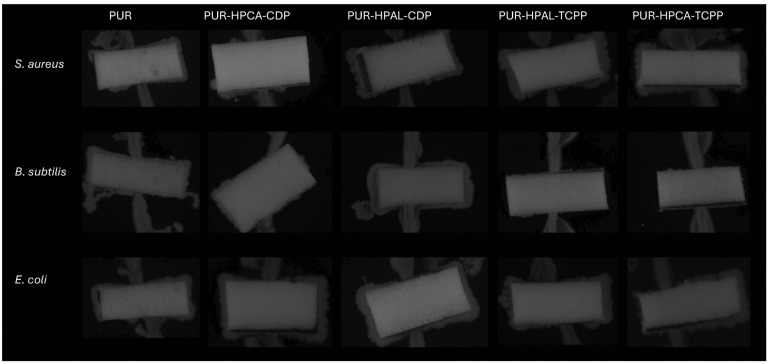
Images of bacterial cultures in the presence of PUR composites.

**Figure 9:**
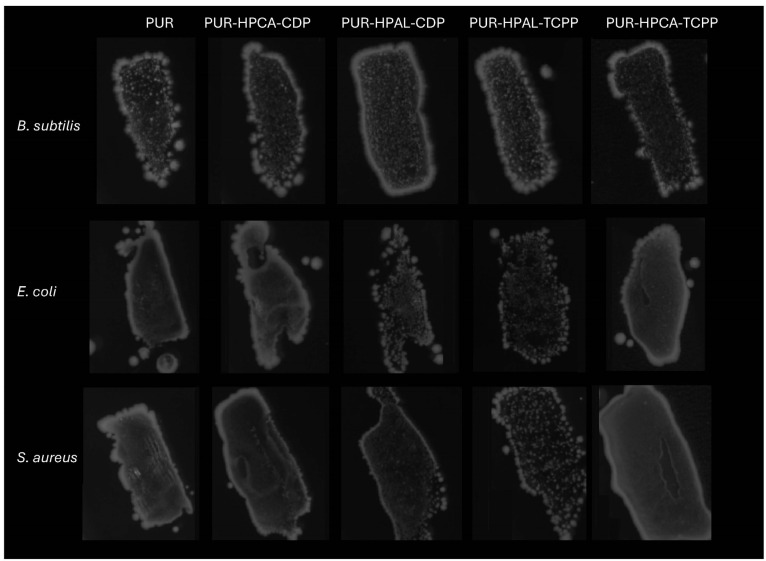
Images of imprints of PUR composites covered with live bacteria.

According to the research assumptions, the developed synergistic systems are intended to be biocompatible for everyday use. Therefore, microscopic observations of bacterial cultures with PUR foams were conducted to confirm bacterial survival during the incubation of the bacterial suspension with PUR composites. The results show clear growth areas around the foams (Figure 8). Additionally, the imprint analysis of the foams after incubation also confirmed that the bacteria transferred from the initial suspension to the foam retained their activity (Figure 9). This result indicates the survival of bacteria during interaction with the modified PUR foams. Therefore, it can be concluded that organophosphorus flame retardants in synergistic systems with phosphinates do not exhibit a toxic impact on material–bacteria interactions. This is an important issue for the safety of using modified materials in daily life. Polyurethane foams can thus be in long-term exposure/contact with humans without the risk of adverse reactions.

## 4. Conclusions

The article presented the use of organophosphorus compounds (CDP and TCPP) in synergistic systems with phosphinate compounds (HPAL, HPCA) in soft polyurethane foam matrices. The obtained results showed increased thermal stability, dm/dt and P_TD_ parameters, and reduced flammability of PUR composites. The results for smoke emission and gaseous, toxic decomposition products are ambiguous. On one hand, the flame retardant systems used in the form of PUR-HPCA-CDP and PUR-HPCA-TCPP reduce the concentrations of CO and HCN in gaseous decomposition products, while on the other hand, they significantly increase the concentration of CO_2_, NOx, and smoke emission. The increase in smoke emission and the CIT_G_ toxicometric index value directly result from the action mechanism of organophosphorus compounds. These compounds, by catalyzing the charring reaction, contribute to increased smoke emission and CO_2_ concentration in gaseous decomposition products, especially at high temperature values, i.e., above 420 °C. Additionally, microbiological studies show that despite the purely chemical modification, the obtained foam material is completely safe to use and does not exhibit biocidal properties.

## Figures and Tables

**Table 1:** Composition of the tested composites (parts by weight).

Composite	HPCA	HPAL	CDP	TCPP
PUR-HPCA-CDP	10		25	
PUR-HPAL-CDP		10	25	
PUR-HPAL-TCPP		10		25
PUR-HPCA-TCPP	10			25

**Table 3:** Results of the thermal stability parameters of PUR composites.

Composite	T_5_ (°C)	T_50_ (°C)	T_RMAX_ (°C)	dm/dt (%/min)	P_TD_ (%)	∆T_s_ (°C)	P_600_ (%)
PUR	280	344	330	11	27.4	450–600	1.55
PUR-HPCA-CDP	260	346	345	10.3	30.6	440–600	4.88
PUR-HPAL-CDP	261	348	346	10.1	34.1	445–600	10.08
PUR-HPAL-TCPP	202	328	325	9.5	34.2	430–600	6.18
PUR-HPCA-TCPP	210	325	323	8.8	32.2	450–600	7.49

**Table 4:** The flammability parameters of PUR composites obtained with the PCFC microcalorimetry method.

Composite	HRR_MAX_ (W/g)	THRR_MAX_ (°C)	THR (kJ/g)	HRC (J/gK)
PUR	496.9	407.9	25.6	462
PUR-HPCA-CDP	274.7	415.2	19.5	256
PUR-HPAL-CDP	312.6	414.2	19.7	300
PUR-HPAL-TCPP	307	423.1	15.6	285
PUR-HPCA-TCPP	283.8	418.2	17	264

**Table 5:** The flammability parameters of PUR composites obtained with the cone calorimetry method.

Composite	PUR	PUR-HPCA-CDP	PUR-HPAL-CDP	PUR-HPAL-TCPP	PUR-HPCA-TCPP
t_i_ (s)	28	37	49	37	37
t_f-o_ (s)	303	176	239	204	207
HRR (kW/m^2^)	44.18	72.53	63.16	57.37	57.09
HRR_max_ (kW/m^2^)	119.9	113.25	122.4	104.33	109.89
THR (MJ/m^2^)	12.2	10.0	12.0	9.4	9.7
EHC (MJ/kg)	12.68	11.18	11.63	10.70	11.03
EHC_max_ (MJ/kg)	39.60	67.19	42.32	62.93	58.89
MLR (g/s)	0.031	0.057	0.048	0.047	0.045
MLR_max_ (g/s)	0.146	0.153	0.144	0.140	0.160
AMLR (g/m^2^·s)	6.14	10.41	10.44	10.16	11.77
FIGRA (kW/m^2^·s)	1.33	1.13	0.98	1.04	1.16
MARHE (kW/m^2^)	61.03	57.48	57.69	53.02	52.44

**Table 6:** Smoke density of PUR composites.

Composite	D_sMAX_	TD_sMAX_	Ds(4)	VOF_4_
PUR	300.1	600	109.4	211.3
PUR-HPCA-CDP	423.2	584	219.9	414.4
PUR-HPAL-CDP	343.6	595	144.4	229.4
PUR-HPAL-TCPP	325.9	584	171.8	335.4
PUR-HPCA-TCPP	271.4	593	158.7	270.6

**Table 7:** Toxic gas emissions during thermal decomposition, toxicometric index CIT_G_.

**Emission in Real Time (ppm)**	**PUR**	**PUR-HPCA-CDP**	**PUR-HPAL-CDP**	**PUR-HPAL-TCPP**	**PUR-HPCA-TCPP**
CO	112,057	90,144	86,023	88,557	88,383
CO_2_	139,806	141,826	220,096	168,757	107,968
HCl	-	-	-	-	-
HCN	7178	6376	5667	6133	6884
SO_2_	-	-	-	-	-
HBr	-	-	-	-	-
HF	-	-	-	-	-
NOx	6564	14,007	12,793	12,527	7810
**Concentration in real time (g/m^2^)**	**PUR**	**PUR-HPCA-CDP**	**PUR-HPAL-CDP**	**PUR-HPAL-TCPP**	**PUR-HPCA-TCPP**
CO	80.9	65.1	62.1	63.9	63.8
CO_2_	158.6	160.8	249.6	191.4	122.4
HCl	-	-	-	-	-
HCN	5.0	4.4	3.9	4.3	4.8
SO_2_	-	-	-	-	-
HBr	-	-	-	-	-
HF	-	-	-	-	-
NOx	7.8	16.6	15.2	14.9	9.3
**CIT_G_**	**PUR**	**PUR-HPCA-CDP**	**PUR-HPAL-CDP**	**PUR-HPAL-TCPP**	**PUR-HPCA-TCPP**
CO	0.36	0.30	0.28	0.29	0.30
CO_2_	0.02	0.02	0.02	0.02	0.01
HCl	-	-	-	-	-
HCN	0.58	0.64	0.60	0.63	0.63
SO_2_	-	-	-	-	-
HBr	-	-	-	-	-
HF	-	-	-	-	-
NOx	1.65	2.26	2.01	1.93	1.48
SUM	2.62	3.22	2.91	2.86	2.41

**Table 8:** Level of bacterial adsorption to the surface of PUR composites obtained by the UV-VIS method.

Composite/Bacteria	*E. coli*	*B. subtilis*	*S. aureus*	Average
PUR	50.5%	45.5%	51%	49%
PUR-HPCA-CDP	52.5%	59.1%	53.1%	55%
PUR-HPAL-CDP	52%	61.6%	57.7%	57.1%
PUR-HPAL-TCPP	56%	52.5%	55.2%	54.6%
PUR-HPCA-TCPP	47.5%	47%	50.5%	48%

## Data Availability

No new data were created or analyzed in this study. Data sharing is not applicable to this article.
